# High‐fat diet‐induced obesity augments the deleterious effects of estrogen deficiency on bone: Evidence from ovariectomized mice

**DOI:** 10.1111/acel.13726

**Published:** 2022-10-10

**Authors:** Dalia Ali, Florence Figeac, Atenisa Caci, Nicholas Ditzel, Clarissa Schmal, Greet Kerckhofs, Jesper Havelund, Nils Færgeman, Alexander Rauch, Michaela Tencerova, Moustapha Kassem

**Affiliations:** ^1^ Department of Endocrinology and Metabolism, Molecular Endocrinology & Stem Cell Research Unit (KMEB) Odense University Hospital University of Southern Denmark Odense Denmark; ^2^ Biomechanics Section, Department of Mechanical Engineering KU Leuven Heverlee Belgium; ^3^ Department of Biochemistry and Molecular Biology, VILLUM Center for Bioanalytical Sciences University of Southern Denmark Odense Denmark; ^4^ Steno Diabetes Center Odense Odense University Hospital Odense Denmark; ^5^ Molecular Physiology of Bone, Institute of Physiology Czech Academy of Sciences Prague Czech Republic; ^6^ Department of Cellular and Molecular Medicine, Danish Stem Cell Centre (DanStem) University of Copenhagen Copenhagen Denmark

**Keywords:** accelerated aging, Aging, bone fragility, bone marrow adiposity, menopause, obesity, osteoporosis, senescence

## Abstract

Several epidemiological studies have suggested that obesity complicated with insulin resistance and type 2 diabetes exerts deleterious effects on the skeleton. While obesity coexists with estrogen deficiency in postmenopausal women, their combined effects on the skeleton are poorly studied. Thus, we investigated the impact of high‐fat diet (HFD) on bone and metabolism of ovariectomized (OVX) female mice (C57BL/6J). OVX or sham operated mice were fed either HFD (60%fat) or normal diet (10%fat) for 12 weeks. HFD‐OVX group exhibited pronounced increase in body weight (~86% in HFD and ~122% in HFD‐OVX, *p* < 0.0005) and impaired glucose tolerance. Bone microCT‐scanning revealed a pronounced decrease in trabecular bone volume/total volume (BV/TV) (−15.6 ± 0.48% in HFD and −37.5 ± 0.235% in HFD‐OVX, *p* < 0.005) and expansion of bone marrow adipose tissue (BMAT; +60.7 ± 9.9% in HFD vs. +79.5 ± 5.86% in HFD‐OVX, *p* < 0.005). Mechanistically, HFD‐OVX treatment led to upregulation of genes markers of senescence, bone resorption, adipogenesis, inflammation, downregulation of gene markers of bone formation and bone development. Similarly, HFD‐OVX treatment resulted in significant changes in bone tissue levels of purine/pyrimidine and Glutamate metabolisms, known to play a regulatory role in bone metabolism. Obesity and estrogen deficiency exert combined deleterious effects on bone resulting in accelerated cellular senescence, expansion of BMAT and impaired bone formation leading to decreased bone mass. Our results suggest that obesity may increase bone fragility in postmenopausal women.

## Graphical abstract

Obesity in female mice, when combined with estrogen deficiency, has a detrimental impact on bone quality, caused by expansion of bone marrow adiposity, upregulation of pro‐inflammatory markers, upregulation of senescence‐associated phenotype markers and accumulation of senescent cells. This also resulted in downregulation of anabolic bone formation pathways, osteoporotic bone phenotype, and increased fracture risk.

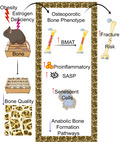

## 1 INTRODUCTION

Postmenopausal estrogen deficiency is a major risk factor for bone fragility and osteoporotic fractures (Kassem & Marie, 2011). Additional risk factors for osteoporosis and fragility fractures include age, parental osteoporotic fractures, medications, for example, glucocorticoids (Buckley et al., 2017) and more recently recognized obesity and its metabolic complication of type 2 diabetes (Kozakowski et al., 2017). This seems paradoxical since obesity is usually associated with increased bone mass (Reid, 2006). However, obesity leads to several complications including impaired macronutrient metabolism (Singla et al., 2010), cardiovascular diseases (Van Gaal et al., 2006) and muscle atrophy that predispose to bone fragility fractures (Proietto, 2020). The relative contribution of these factors cannot be resolved using human epidemiological studies and can be studied using experimental animal models.

We have previously demonstrated that diet‐induced obesity in male mice resulted in decreased bone mass associated with accelerated senescence and impaired differentiation of skeletal stem cells (also known as marrow stromal cells, BMSCs) which we suggested as potential mechanism for obesity‐induced bone fragility (Tencerova et al., 2018). However, the effects of obesity on female animal skeleton are poorly studied. Only one previous study in mice has reported that obesity was not protective against OVX‐mediated bone loss, but the study was descriptive and did not provide a plausible mechanism (Cao & Gregoire, 2016). In humans, postmenopausal state is associated with increased prevalence of obesity due to changes in energy metabolism (reviewed in Ko and Kim (2020)) and obesity complications of increased risk for type 2 diabetes and cardiovascular complications (Tandon et al., 2010). Human studies of the effect of obesity in the postmenopausal women on risk of fractures yielded variable results. Some studies reported that obese postmenopausal women have lower risk for osteoporotic fractures (Andreoli et al., 2011; Heidari et al., 2015; Migliaccio et al., 2011; Silva et al., 2007; Yanik et al., 2009), while other studies reported increased fracture risk (Compston et al., 2011; Greco et al., 2010; Rikkonen et al., 2021) reflecting the heterogeneity of the populations studied and the limitation of using epidemiological methods to correct for confounding factors.

There is an increasing interest in studying regulatory role of bone marrow adipose tissue (BMAT) on bone remodeling. and age‐related expansion of BMAT has been proposed as a causative factor leading to age‐related bone loss (Nehlin et al., 2019). Estrogen deficiency in postmenopausal period has been associated with increased BMAT (Syed et al., 2010). We and others have shown that diet‐induced obesity in mice leads to expansion of BMAT in male mice (Scheller et al., 2016; Tencerova et al., 2018). However, the combined effects of obesity and estrogen deficiency in female mice and humans are not known. The cellular mechanisms underlying BMAT expansion are thought to be caused by changes in the BMSCs niche leading to a shift in differentiation fate of BMSCs from osteoblasts (OB) to adipocytes (AD; Tencerova & Kassem, 2016; Veldhuis‐Vlug & Rosen, 2018).

Cellular senescence is considered one of the fundamental mechanisms underlying aging (Khosla et al., 2020) and recently has been demonstrated to mediate age‐related bone loss (Farr et al., 2017). Cellular senescence indicate that the cell has lost its proliferative potential leading to irreversible growth arrest and is the result of accumulation of molecular cellular damage (Kumari & Jat, 2021). Despite cell cycle arrest, senescent cells remain viable, and they are capable for producing a unique secretome, termed the senescence‐associated secretory phenotype (SASP), which is comprised of pro‐inflammatory cytokines and chemokines, as well as extracellular matrix degrading enzymes that have deleterious effects on tissue functions (Coppe et al., 2010). While one previous study showed that estrogen deficiency induced by ovariectomy (OVX) leads to increased senescent cell burden (Wu et al., 2020), Farr et al. were not able to corroborate this finding (Farr et al., 2019). On the contrary, we have previously demonstrated that HFD‐induced obesity in male leads to increased burden of senescence cells in bone (Figeac et al., 2022) and in cultured cells (Tencerova et al., 2019) that may contribute to impaired fracture healing and bone fragility observed in obesity. The combined effects of obesity and estrogen deficiency on bone cellular senescence are not known.

In order to study the relative contribution of obesity and estrogen deficiency to changes in the skeleton, we employed an OVX‐mouse model with HFD‐induced obesity as a model for postmenopausal women. Employing cellular, transcriptomic, and metabolic investigations, our results demonstrated that obesity interacts with estrogen deficiency leading to expansion of BMAT and accelerated cellular senescence in the bone and bone marrow microenvironment, resulting in impaired bone formation and decreased bone mass.

## 2 RESULTS

### 2.1 HFD‐OVX reduces bone mass

**FIGURE 1:**
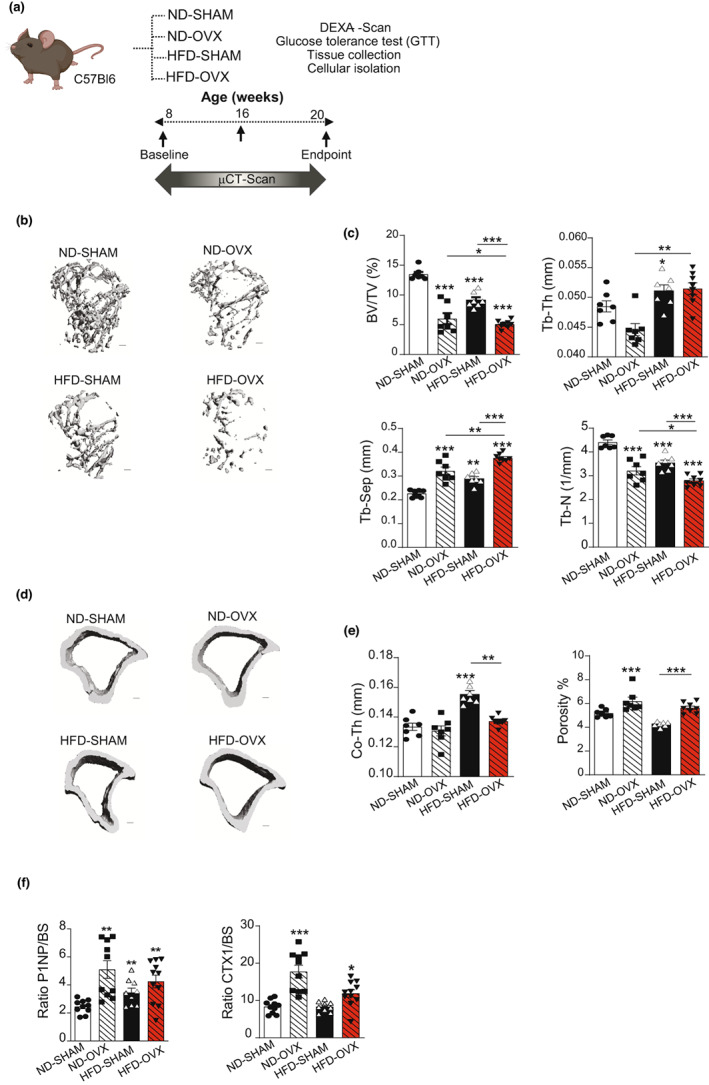
Effect of HFD‐induced obesity on bone parameters. (a) Study design. Bone parameters as evaluated by μCT, 8 weeks after the surgery, (b) Representative images for μCT 3D reconstruction of trabecular compartment (c) trabecular bone parameters were evaluated as bone volume per total volume (BV/TV), trabecular number (Tb.N), trabecular thickness (Tb.Th), and trabecular separation (Tb.Sep). (d) Representative images for μCT 3D reconstruction of cortical compartment. (e) Cortical diaphysis bone measurements as cortical thickness (Co.Th) and porosity %. (f) Markers for bone turnover, ratio P1NP/BS (bone surface‐ mm^2^; *n* = 10 per group) for bone formation (left panel) and ratio CTX‐1/BS (*n* = 9 per group) for bone resorption (right panel) were measured in serum. Data are presented as mean ± SEM (*n* = 7–10 mice/group). One‐way ANOVA, *p < 0.05, **p < 0.005; ***p < 0.0005

Tibiae from animals belonging to the 4 experimental groups, ND‐SHAM, ND‐OVX, HFD‐SHAM, and HFD‐OVX (Figure 1a), were scanned by μCT and changes in bone mass were determined at 8 weeks post‐OVX and compared with ND‐SHAM (Figure 1b–e). The HFD‐OVX group exhibited the most pronounced trabecular bone loss (−62.44% as compared to ND‐SHAM, while −55.4% for ND‐OVX and −32.15% for HFD‐SHAM) while the cortical bone mass was unchanged (Figure 1d,e). The observed trabecular bone phenotype at 8 weeks post‐OVX was maintained at 12 weeks; moreover, at this time point, we observed decreased cortical bone mass in the HFD‐OVX group (Figure S1A–D). Overall, we observed that the combination of obesity and estrogen deficiency at 12 weeks post‐OVX has a more detrimental effect on bone loss both cortical and trabecular (Figures 1b–e and S1A–D). TRAcP evaluation of Osteoclast surface per bone surface (OcS/BS) and Osteoclasts number per bone perimeter (OcN/BPm) did not show significant difference between groups (Figure S1E). Finally biochemical bone turnover markers showed a high bone turnover state following OVX (Yoon et al., 2012), a low bone turnover following HFD (Tencerova et al., 2018) and in HFD‐OVX group an intermediary bone turnover (Figure 1f).

### 2.2 HFD‐OVX leads to obesity and impaired glucose tolerance

**FIGURE 2:**
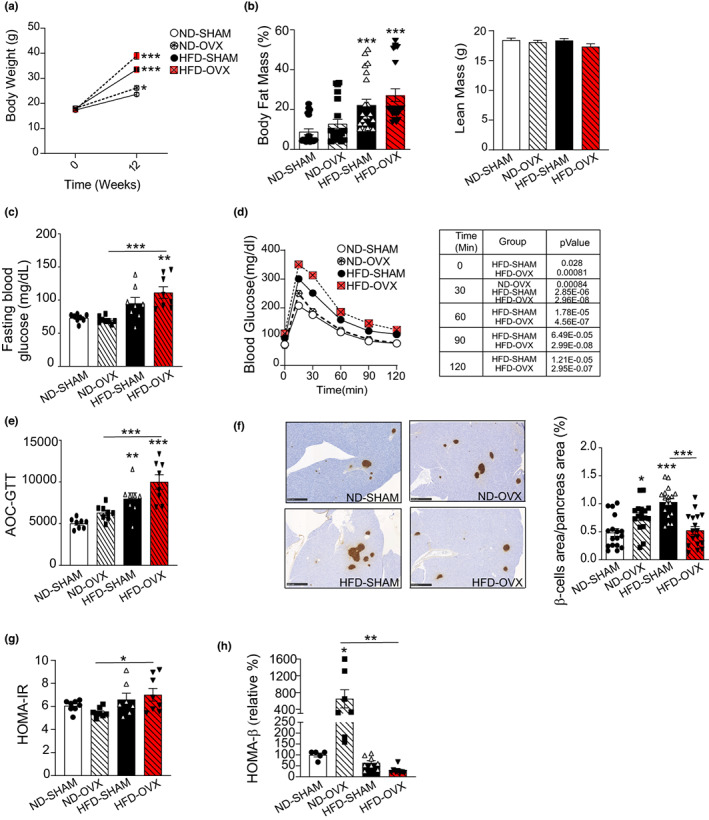
Effect of HFD on body composition and glucose metabolism. Evaluation of the body composition at the endpoint of the study (eight mice/group). (a) Body weight (b) Fat mass percentage and lean mass (absolute value). Glucose metabolism as evaluated by (c) Fasting blood glucose (mg/dl), (d) GTT and (e) area of the curve (AOC). (f) Representative photomicrographs of insulin staining of pancreas of ND‐SHAM, ND‐OVX, HFD‐SHAM &HFD‐OVX mice (left panel). Scale bar: 500 μm. Quantified β‐cell area expressed as percentage of total area (right panel). (g) HOMA‐IR as calculated [(fasting blood glucose (mg/dl) * fasting insulin (mIU/L))/405]. (H) Relative HOMA‐β = 20 × [FI (mIU/L)/(FBG [mmol/L] − 3.5) (%)]. Data are presented as mean ± SEM (*n* = 17 for (a) to (e) and *n* = 5 to 8 for (f) to (h)), *p < 0.05, **p < 0.005; ***p < 0.005; 1‐way ANOVA

During 12 weeks of observation, changes in body weight were dependent on the experimental condition with ~37.5% in ND‐SHAM group, ~45% in ND‐OVX group, ~88% in the HFD‐SHAM group, and ~124.5% in the HFD‐OVX group (Figure 2a). Body fat mass percentage as evaluated by dual‐energy X‐ray absorptiometry (DEXA) scanning exhibited significant difference between the experimental groups: 20.5% in ND‐SHAM, 27.7% in ND‐OVX, 43.4% in HFD‐SHAM, and 51.33% in HFD‐OVX (Figure 2b, left panel) while body lean mass was unchanged between groups (Figure 2b, right panel). Fasting blood glucose (Figure 2c) was increased in both HFD fed groups as compared to ND fed animals and changes in GTT calculated as AOC, revealed a progressive impairment in glucose tolerance from ND‐OVX, HFD to HFD‐OVX (*p* < 0.005; Figure 2d,e). To determine the mechanism, we quantified pancreatic β‐cell mass that was significantly increased by ~28.86% in ND‐OVX, ~54.36% in HFD‐SHAM while decreased in the HFD‐OVX compared with HFD‐SHAM group up to the ND‐SHAM level revealing a β‐cell failure in this group to potentially compensate for a peripheral insulin resistance explaining why the glucose metabolism of this HFD‐OVX group, as visualized by GTT, is the worst (Figure 2f). Moreover, calculation of HOMA‐IR (for insulin resistance) and HOMA‐β (for pancreatic β‐cell function) parameters showed a trend to increased HOMA‐IR value in HFD‐SHAM group that was accentuated in the HFD‐OVX group (Figure 2g) combined with an trend to impaired insulin secretion as evaluated by the HOMA‐β ratio, which was more pronounced in the HFD‐OVX group as compared to the ND‐SHAM group (Figure 2h). Interestingly in our study, we did observe that the ND‐OVX group glucose tolerance through a combination of increased β‐cell area and function (Figure 2f–h).

### 2.3 HFD‐OVX leads to BMAT expansion, inflammation, and insulin resistance in bone

**FIGURE 3:**
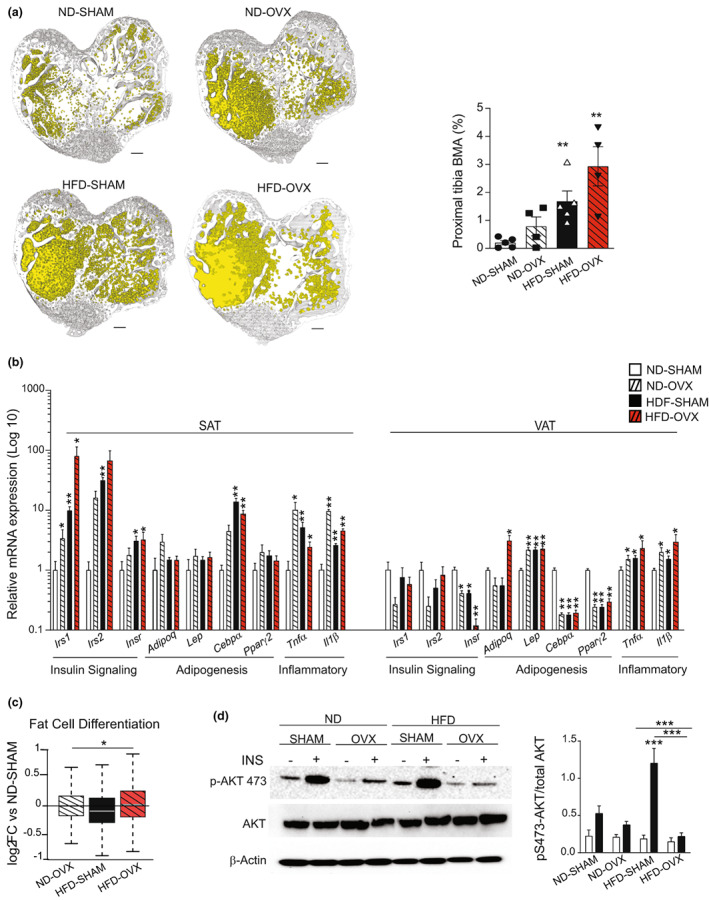
Effect of HFD on bone marrow and peripheral fat depot. (a) Left panel, representative images for μCT‐3D reconstruction of proximal tibia bone marrow adiposity from the side of the primary spongiosa, scale bar = 250 μm. Right panel, quantification of bone marrow adiposity in proximal tibia (%). (b) Quantification of mRNA expression levels of insulin signaling‐related genes (*Irs1*, *Irs2*, *Insr*), adipogenic genes (*Adipoq*, *Cebpα*, *Pparγ2*) and inflammatory genes (*Tnfa*, *Il1β*) in SAT (left panel) and VAT (right panel) (ND‐SHAM (*n* = 6), ND‐OVX (*n* = 8), HFD‐SHAM (*n* = 8) and HFD‐OVX (*n* = 8)). (c) Box plot showing the log fold change in expression levels of genes linked to fat cell differentiation in BMSCs from ND‐OVX, HFD‐SHAM and HFD‐OVX mice compared to control mice ND‐SHAM. Mann‐Whitney test with **p < 0.01. (d) Western blot of AKT, pS473‐AKT and β‐actin and densitometry evaluation of p‐S473‐AKT versus AKT in undifferentiated BMSCs with and without 15 min of insulin stimulation (100 nM). Data are presented as mean ± SEM from three independent experiments (***p < 0.0005)[Correction added on 25 October 2022, after first online publication: the figure caption related to part figure (c) was missing and it has been included in this version.]

Both estrogen deficiency and obesity dependent bone loss have been associated with increase in marrow adiposity. Quantifying BMAT content using μCT scanning (Figure 3a) revealed an increase by ≈289% in ND‐OVX, ≈732% in HFD‐SHAM, and ≈1352% in HFD‐OVX. Obesity‐induced insulin resistance in adipose tissue is known to affect adipocyte function in part through low‐grade chronic inflammation (Reilly & Saltiel, 2017; Zatterale et al., 2019). We quantified mRNA levels of genes related to inflammation (*Tnfα & Il1β*), related to insulin signaling (*Irs1, Irs2 & Insr*), and related to adipogenesis (*Lep, Cebpα, Pparγ2*) in subcutaneous (SAT) and visceral adipose tissue (VAT; Figure 3b). Gene expression profile in VAT revealed a decrease expression of insulin receptor gene but not IRS1 and 2. However, we observed decreased gene expression of adipogenesis‐related genes and increased gene expression of inflammation‐related genes, in HFD‐SHAM, ND‐OVX, and HFD‐OVX groups (Figure 3b). On the contrary, genes related to insulin signaling showed increased expression in SAT (Figure 3b). In line with previously reported data from our group (Tencerova et al., 2018), insulin signaling was maintained if not improved in isolated and cultured bone derived BMSCs from HFD‐SHAM mice (Figure 3c) as evidenced by the phosphoprotein levels of AKT presented as the ratio pAKT‐S473/total AKT. Insulin sensitivity was lost in BMSCs isolated from HFD‐ovariectomized (OVX) mice (Figure 3c).

### 2.4 HFD‐OVX is associated with downregulated genetic pathways of bone formation in BMSCs

**FIGURE 4:**
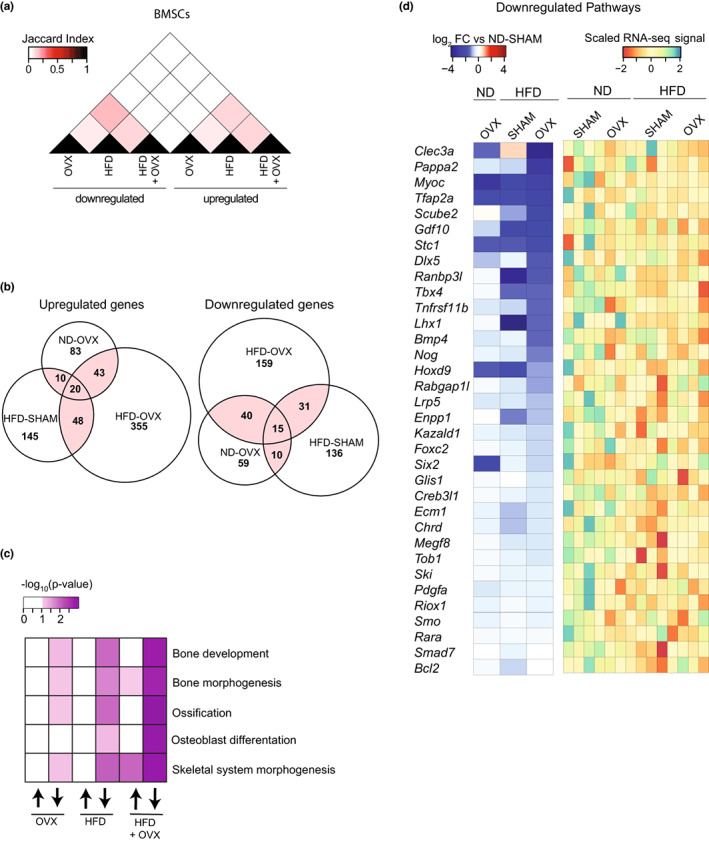
Effect of HFD‐induced obesity and estrogen deficiency on osteogenic molecular signature in BMSCs. (a) Heat map showing a Jaccard index based on the overlap of up‐and downregulated genes (*p* < 0.05) in BMSCs from ND‐OVX, HFD‐SHAM, and HFD‐OVX mice compared to control mice ND‐SHAM. (b) Venn diagram showing the overlap among genes that are up‐or downregulated (*p* < 0.05) in BMSCs from ND‐OVX, HFD‐SHAM, and HFD‐OVX mice compared to control mice ND‐SHAM. (c) Heat map showing the enrichment of gene ontology terms related to bone development, bone morphogenesis, ossification, osteoblast differentiation and skeletal system morphogenesis among the up and down‐regulated genes in BMSCs from ND‐OVX, HFD‐SHAM, and HFD‐OVX mice compared to control mice ND‐SHAM. (d) Heat map showing the log fold change of genes from the indicated gene ontology pathways

To identify molecular mechanisms mediating bone loss in HFD‐OVX, we evaluated mRNA levels of selected osteoblastic, osteoclastic, and adipocytic gene markers using mRNA isolated from whole bone (Figure S2A,B), or whole bone marrow (Figure S2C,D). HFD‐OVX was associated with increase in adipocytic genes: *Adipoq*, *Cebpα*, *Lpl*, *Fabp4*, *Pparγ2* and in *Alpl* and *Tnfsf11* (encoding RANKL) mRNA levels (Figure S2A–D), which was less prominent in the HFD‐SHAM and ND‐OVX groups. Those increased transcript levels of osteoblast and osteoclast activity partly match the elevated bone turnover (Figure 1f); however, the ND‐OVX with the strongest increase in bone turnover showed the lowest induction levels of *Alpl* and *Tnfsf11*. We focused on isolated BMSCs to test whether the altered microenvironment in the bone of ND‐OVX, HFD‐SHAM, or HFD‐OVX mice induces epigenetic changes that maintain an altered expression profile linked to the deteriorated bone phenotype (Figure S3A–C). Comparative overlaps shows that the three treatment conditions do not lead to changes in opposing directions, but rather have similar changes or group‐specific regulation patterns (Figure 4a,b). Clustering of the differentially expressed genes revealed patterns that are specific to HFD, OVX, as well as the combined treatment (Figure S4A,B) and that differ in their enrichment for signaling pathways and gene ontology terms (Figure S4C,D). GO pathway analysis revealed that downregulated genes from HFD‐OVX BMSCs are associated with bone development, bone morphogenesis, ossification, osteoblast differentiation, and skeletal system morphogenesis (Figure S3C) which is less prominent in ND‐OVX and HFD‐SHAM conditions (Figure 4c). Comparing this group of genes across all experimental conditions, we observed that the strong downregulation in HFD‐OVX is partly recapitulated by the single treatments but often due to an additive effect of both (Figure 4d).

### 2.5 HFD‐OVX leads to accelerated senescence in the bone and bone marrow microenvironment

**FIGURE 5:**
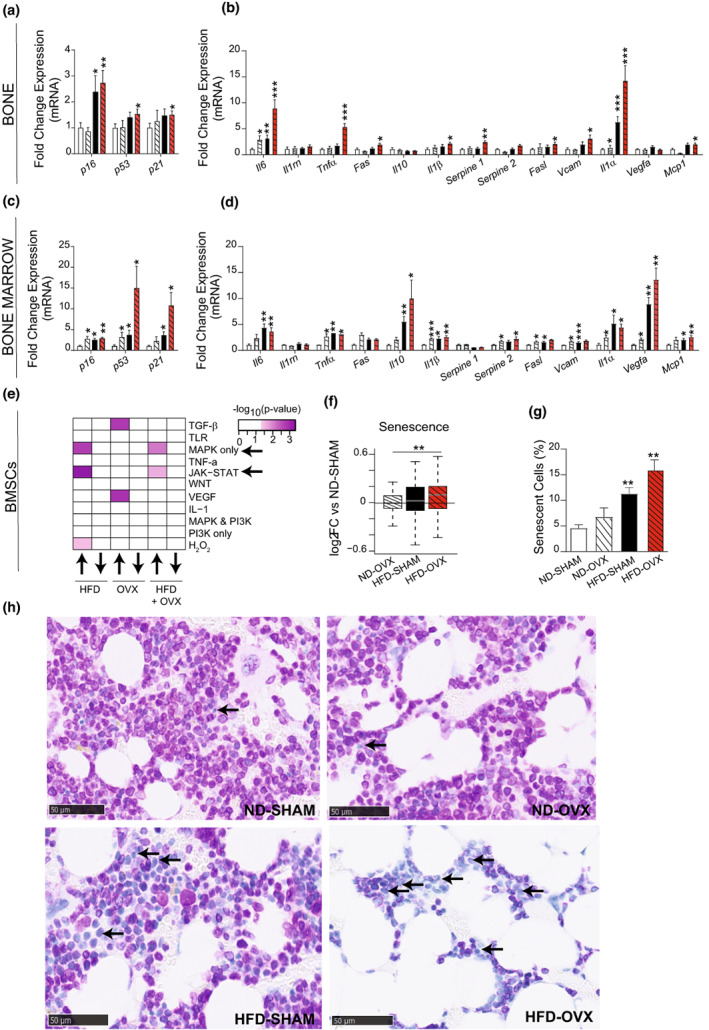
HFD‐induced obesity upregulated senescence in bone, bone marrow and BMSCs. Quantification of mRNA expression levels using qPCR (*n* = 10) of (a, c) senescence‐associated markers and (b, d) SASP markers in bone (a, b) and bone marrow (c, d) of ND‐SHAM, ND‐OVX, HFD‐SHAM, and HFD‐OVX mice at the end of the experiment. (e) Heat map showing enrichment analysis of genes, up and down regulated, in BMSCs of ND‐OVX, HFD‐SHAM, and HFD‐OVX mice that are under control of the indicated pathways (SPEED pathway analysis). (f) Box plot showing the log fold change in expression levels of genes linked to senescence in BMSCs from ND‐OVX, HFD‐SHAM, and HFD‐OVX mice compared to control mice ND‐SHAM. Mann–Whitney test with **p < 0.01. (g) Senescent cells percentage (cells not immunostained for LMNB1). (h) Immunostaining for Lamin B1 (LMNB1; a negative marker of cellular senescence) performed on tibial sections. Lack of staining is indicating senescent cells (black arrow). Scale bar: 50 μm. Data are presented as mean ± SEM, *p < 0.05, **p < 0.005; ***p < 0.0005. Two‐tailed unpaired Student's *t*‐test and 1‐way ANOVA.

We have previously reported that in male mice and humans, obesity is associated with an accelerated senescence phenotype in cultured BMSCs (Figeac et al., 2022; Tencerova et al., 2019).Thus, we investigated gene expression profile of senescence‐associated genes in bone and bone marrow samples. HFD‐OVX exhibited higher expression levels of senescence‐associated genes (p16, p53 and p21; Figure 5a,c) and the SASP marker genes (Figure 5b,d). In addition, RNA‐seq of cultured BMSCs revealed enrichment for target genes of MAPK and JAK–STAT signaling in HFD‐OVX (Figure 5e), known to be associated with cellular senescence (Anerillas et al., 2020; Farr et al., 2017; Ji et al., 2017; Xu et al., 2016) as well as a general increase of genes from the GO term “senescence pathway” (Figure 5f). Finally, immunostaining for Lamin B1 (LMNB1; a negative marker of cellular senescence; Baar et al., 2017; Figeac et al., 2022) performed on sections from proximal tibia showed increased number of senescent cells in HFD‐SHAM and a more pronounced effect in the HFD‐OVX (Figure 5g,h). Interestingly, OVX alone did not result in increased number of senescent cells suggesting that obesity is the main driving force for the accelerated senescence phenotype.

### 2.6 Impact of HFD and estrogen deficiency on metabolic profiling of femur

**FIGURE 6:**
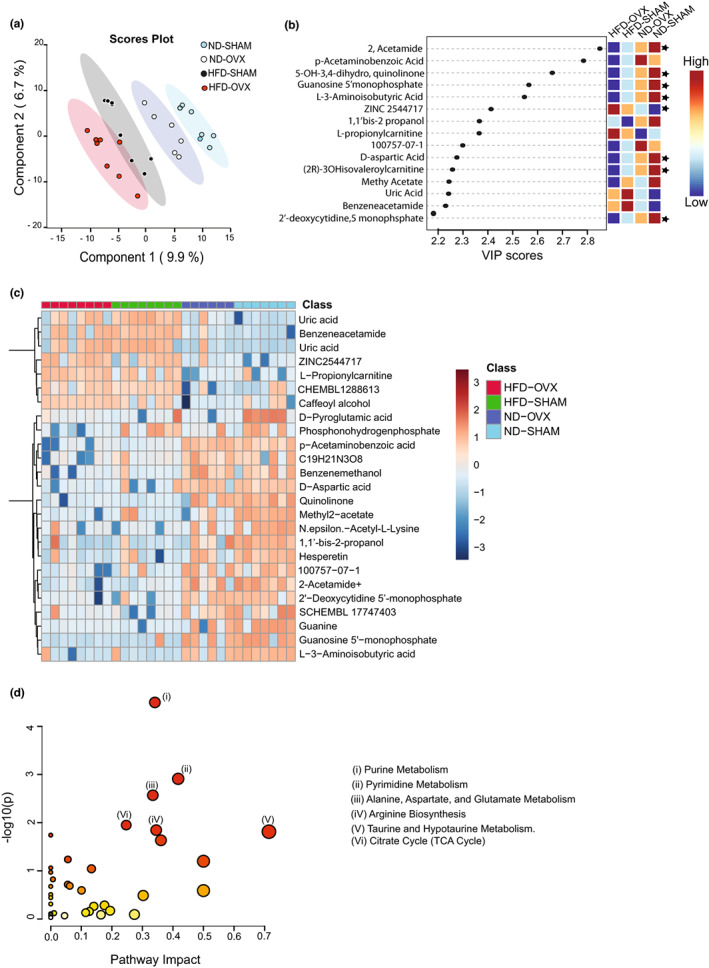
Full bone metabolic profiling. Composition in metabolites of full bones from ND‐SHAM, ND‐OVX, HFD‐SHAM, and HFD‐OVX groups. Global metabolomic analyses of metabolites from full femurs using liquid chromatography‐mass spectrometry (LC–MS). (a) PLS‐DA score plot of the ND‐SHAM, ND‐OVX, HFD‐SHAM, and HFD‐OVX groups in metabolomic analysis. (b) Variable importance in projection (VIP) from PLS‐DA analysis, colored boxes on the right indicate the relative concentrations of the corresponding metabolites in each group under study (black arrows are indicating those with gradual variation following the modelization pattern suggested by the PLS‐DA). (c) Heat map of the top 25 metabolites that are differentially expressed in the full bones of ND‐SHAM, ND‐OVX, HFD‐SHAM, and HFD‐OVX mice. (d) Metabolic pathway analysis. Matched pathways are displayed as circles and color and size of each circle are based on *p* value and pathway impact value, respectively. The pathways with the highest statistical significance scores are indicated as follows: (i) Purine metabolism, (ii) Pyrimidine metabolism, (iii) Alanine, Aspartate and Glutamate metabolism, (iv) Arginine biosynthesis, (v) Taurine and Hypotaurine metabolism, and (vi) TCA cycle

Changes in intermediary metabolism provides a link between hormonal changes in microenvironment and cellular phenotype (Ferreira et al., 2020; Muller & Seitz, 1984). We performed a global metabolomic analysis of crushed femurs obtained from ND‐SHAM, ND‐OVX, HFD‐SHAM, and HFD‐OVX using liquid chromatography‐mass spectrometry (LC–MS; Figure 6). While principal component analysis was capable to detect variation based on diet but not surgery (Figure S5A), partial least square discriminant analysis (PLS‐DA; Figure 6a) showed a clear separation of the treatment groups. The internal validity of the model was evaluated by estimating *R* ^2^ and *Q* ^2^ parameters that were both above the threshold for a significant biological model (Psihogios et al., 2008; Figure S5B). From PLS‐DA analysis, we also observed that eight out of 15 top metabolites based on VIP scores that exhibited significant changes within experimental groups, followed gradual changes from ND‐SHAM over ND‐OVX and HFD‐SHAM to HFD‐OVX (Figure 6b). ANOVA analysis of our dataset provided a list of 12 metabolites with significantly different concentration between the experimental groups (Table S1) and revealed that purine/pyrimidine as well as carbohydrate and lipid metabolisms explain the observed differences among the experimental groups. The heatmap based on the comparison of the levels of metabolites in all the experimental groups, confirmed that the HFD was the driving force for the observed differences in metabolite concentration and metabolic pathways, among the experimental groups (Figure 6c). Among those metabolites, many were linked to purine/pyrimidine as well as glutamate metabolism (Figure 6d).

## 3 DISCUSSION

In this study, we investigated the combined effects of HFD‐induced obesity and estrogen deficiency on bone and BMAT in female mice as a model for obese postmenopausal women. We also determined the metabolic and molecular mechanisms underlying the observed changes and we demonstrated that obesity and estrogen deficiency heighten the negative effects of obesity or estrogen deficiency alone, on bone and bone marrow microenvironment and led to severe deleterious effects on the skeleton.

As expected, 12 weeks of HFD feeding led to obesity along with impairment of glucose metabolism in female mice and these changes were aggravated when combined with estrogen deficiency. Our findings in female mice are similar to previous studies in male mice that showed negative impact of HFD on glucose tolerance (Gallou‐Kabani et al., 2007; Tencerova et al., 2018; Winzell & Ahrén, 2004) and similar to the one study that combined HFD with OVX (Gorres‐Martens et al., 2018). Interestingly, we observed that OVX mice maintained glucose tolerance by increasing β cell area and function. Similar findings were reported in a recent study on OVX rats (Chen et al., 2021). Furthermore, the impaired glucose tolerance observed in HFD‐SHAM was partially caused by diminished capacity of the β cell to secrete insulin even though β cell area was increased as evidenced by HOMA‐β. This mechanism was more pronounced in the HFD‐OVX where a combination of decreased β‐cell area and β‐cell function were observed in addition to the presence of insulin resistance suggesting that the compensatory mechanisms of enhanced β cell functions by OVX are compromised by HFD. Direct measurements of insulin levels and performing glucose‐simulate‐insulin‐secretion test needs to be conducted to support these observations.

Like previous studies performed in male mice, the observed decreased bone mass caused by HFD alone in female mice was similar in direction but quantitatively milder (Tencerova et al., 2018). Our results are also similar to the findings of deteriorated bone microstructure, and increased bone fragility observed in male mice (Cao et al., 2009; Devlin et al., 2018; Fujita et al., 2012; Gautam et al., 2014; Kyung et al., 2009; Patsch et al., 2011; Scheller et al., 2016; Shu et al., 2015; Tencerova et al., 2018). We observed that combined HFD and OVX exerted a significant decreased both trabecular and cortical bone mass, compared with OVX or HFD alone which is similar to two studies where OVX performed at age of 6–12 weeks and the mice received 10–18 weeks of HFD (Cao & Gregoire, 2016; Ludgero‐Correia Jr. et al., 2012; Núñez et al., 2007).

OVX‐associated bone loss is a very well‐studied phenomenon, and it is caused by multiple mechanisms but most importantly upregulation of pro‐inflammatory cytokines including RANKL within the bone microenvironment leading to enhanced osteoclastogenesis (Georgiou et al., 2012; Khosla & Pacifici, 2013). We observed significant effects of estrogen deficiency on senescent cell formation only in combination with obesity. This applied to the number of senescent cells formed and the expression of SASP genes. Our findings corroborate previous results reported by Farr et al. that did not detect an independent contribution of estrogen deficiency on senescent cell formation in mice (Farr et al., 2019). However, the observed interaction between obesity and estrogen deficiency suggests that both conditions target senescence‐associated genetic pathways. Cellular senescence is known to enhance osteoclastogenesis and impair osteoblastogenesis leading to bone loss (Farr et al., 2016, 2017). We did not measure histologically the cellular activities of osteoclastic and osteoblastic cells. Alternatively, our genetic studies support this notion as they revealed an upregulation of MAPK and JAK–STAT target genes which are two signaling pathways involved in the regulation of cellular senescence and bone cell functions. MAPK signaling activates expression of pro‐inflammatory molecules and senescence‐associated factors in the p21/p53 and p16/RB pathways (Anerillas et al., 2020) while JAK–STAT signaling regulate SASP production since many of the SASP genes contain JAK–STAT responsive elements (Farr et al., 2017; Ji et al., 2017; Xu et al., 2016).

We observed increased BMAT mass in HFD obese mice which was more pronounced when combined with estrogen deficiency. Increased in BMAT in HFD mice has been reported previously in male mice (Scheller et al., 2016; Tencerova et al., 2018) while estrogen deficiency has been reported to increase BMAT in mice (Elbaz et al., 2009; Georgiou et al., 2012) and humans (Veldhuis‐Vlug & Rosen, 2018). In our study, the increased BMAT was associated with decreased bone mass. However, Almeida et al. (2020) reported that increased BMAT was not associated with appendicular bone loss in female mice. Other studies reported that increased BMAT in obese mice is associated with decreased bone mass (Ambrosi et al., 2017). It is plausible that the observed association between expansion of BMAT and decreased bone mass in HFD‐OVX mice may be casual for the following reasons. First, expansion of BMAT is usually the result of changes in lineage allocation and a shift in the differentiation fate of BMSCs from OB to AD which results in recruitment of fewer osteoblastic cells and impaired bone formation (Nehlin et al., 2019). Second, there exists a crosstalk between BMAT and extra‐medullary adipose tissue depots that leads to bone loss. For example, a recent clinical study reported a positive relationship between BMAT and visceral fat and an inverse relation with bone formation and bone mass in postmenopausal women (Bredella et al., 2011). In our study, visceral obesity was associated with upregulated expression of pro‐inflammatory cytokines, for example, TNFα and IL1β which leads to a state of chronic sterile inflammation and high expression of RANKL enhancing the formation and functions of osteoclastic cells and bone resorption (Khosla, 2001). In one previous study, HFD‐induced obesity has been reported to enhance ovariectomy‐associated inflammatory response (Ludgero‐Correia et al., 2012) which corroborates this hypothesis.

Is there a link between BMAT expansion and the creation of senescent microenvironment observed in combined obesity and estrogen deficiency? The observed increase in BMAT may be caused, by enhanced responsiveness of BMSCs to insulin leading to increased formation of AD as shown in our studies (Tencerova et al., 2018, 2019). In our previous study in male mice (Tencerova et al., 2018) and in the current study, obesity‐associated enhanced insulin signaling in BMSCs created a state of cellular hypermetabolism with increased mitochondrial oxidative phosphorylation (OXPHOS), and levels of reactive oxygen species that can lead to DNA damage and cellular senescence (Tencerova et al., 2019). Interestingly, we observed that OVX alone impairs insulin signaling and this effect persisted in combined OVX‐HFD suggesting that OVX‐associated enhancement of cellular senescence is related to an insulin‐independent mechanism, for example, enhanced inflammatory responses (Freund et al., 2010; Ren et al., 2009) or direct effects on senescence‐associated genes (Farr & Khosla, 2019; Li et al., 2020).

Studying the metabolomic changes taking place in bone of HFD‐OVX revealed additional mechanism that may explain the negative combined effects of obesity and estrogen deficiency on bone mass. However, it is to note that based on molecular biology and metabolomic analyses, obesity (HFD) seems to be the driving force of the observed cellular inflammation and senescence. The metabolomic profile present in bones of HFD‐OVX showed significant changes in the metabolites belonging to purine and pyrimidine, carbohydrate and lipids and glutamate pathways. Particularly, the decrease in metabolites from purine and pyrimidine pathways, may explain the observed increased in cellular inflammation, as it has been reported that pyrimidine and its derivatives exert anti‐inflammatory as well as antioxidant effects, and inhibit osteoclastogenesis and bone resorption via RANKL inhibition (Ihn et al., 2017; Mitsuya & Broder, 1986; Xie et al., 2009). Also, we observed a decrease in metabolites from the glutamate pathway, known to be important for BMSCs stemness and bone homeostasis (Zhou et al., 2019).

Our study has some limitations. We started our HFD intervention at relatively younger age (8 weeks old mice). At this early age, the mice did not reach skeletal maturity which may explain the lower bone mass phenotype observed in these mice which is at variance with the observed normal or increased bone mass observed in obese adult humans (Burghardt et al., 2010; Reid, 2006; Starr et al., 2018). Regarding the metabolic studies, our results are confounded by differences in the macronutrient composition and the source of macronutrients between ND and HFD with respect to fat, protein and carbohydrates that may have affected our results, and thus, those findings are preliminary and should be confirmed in futures studies.

Our findings are relevant to human physiology as the metabolic and hormonal changes observed in HFD and OVX mice parallel known changes in humans (Szmuilowicz et al., 2009; Zhu et al., 2013) and provide insight into the mechanisms underlying the interaction between obesity and estrogen deficiency, an area of research that has been poorly studied. While our study results need to be validated in human females, it suggests a more aggressive intervention strategy in postmenopausal obese women to correct hormonal and metabolic changes in order to maintain and improve bone health.

## 4 EXPERIMENTAL PROCEDURES

### 4.1 Animal model

Female C57BL/6J mice (Taconic) at 8 weeks of age were assigned for either ovariectomy (OVX) or sham operation and afterwards fed a R‐70 normal diet (ND; Lantmännen; containing Kcal%: protein 14.5%, carbohydrates 60%, and fat 4.5%) in which fat content was from Oatmeal, barley, wheat bran, wheat flour (diet details shown in Table S2), or high‐fat diet (HFD; Research Diet D12492, containing Kcal%: protein 20%, carbohydrates 20%, and fat 60%) in which fat content was from soybean oil and lard (diet details shown in Table S3). Mice were anesthetized before ovariectomy by the dorsal approach using intraperitoneal injection of ketamine (100 mg/kg) and xylazine (10 mg/kg). Animals were fed ad libitum and housed (four animals per cage) under standard conditions (21°C, 55% relative humidity) on a 12‐h light/dark cycle for a period of 12 weeks. All experimental procedures were approved by the Danish Animal Ethical committee (2017‐15‐0201‐01210).

### 4.2 Glucose tolerance test (GTT)

Glucose tolerance test (GTT) was performed after 12 weeks of diet intervention. For GTT, overnight‐fasted mice were injected with D‐glucose 1 g/kg i.p. and blood glucose levels were measured from the tail tip at different timepoints using a CONTOUR® Glucometer (Tencerova et al., 2018).

### 4.3 Bone mass measurement via DEXA scanning

Total body fat and lean mass percentage were evaluated using DEXA by using a PIXImus2® (version 1.44; Lunar Cooperation). DEXA scans were acquired after sedating mice with isoflurane at the end of the experiment.

### 4.4 Insulin ELISA

Mouse Insulin ELISA (Abcam: ab277390) kit was used to measure insulin level in serum samples from fasted animals at the end of the experiment.

### 4.5 Histology and immunohistochemistry

Tibias and pancreases were fixed with 4% formalin for 2 days and tibias were demineralized in phosphate buffered saline (PBS)‐EDTA 12% for 14 days. Tissues were embedded in paraffin and sections were used for hematoxylin and eosin staining or immunostaining. In brief, paraffin tissue sections were deparaffinized, dehydrated, and heat treated before incubation with primary antibody against insulin (DAKO A0564) and Lamin B1 (abcam ab16048) followed by secondary antibody, chromogen visualization and counterstaining with hematoxylin. Insulin immunostaining was used to evaluate β‐cell area per tissue area in at least 4 non‐consecutive pancreas sections, and senescent cells in bone were counted as Lamin B1negative cells in bone sections (Baar et al., 2017; Figeac et al., 2022; a minimum of 1500 cells were evaluated for each condition).

### 4.6 Micro‐computed tomography scanning (μCT)

#### 4.6.1 Bone parameters

Proximal tibias of mice fed for 8 and 12 weeks with HFD or with ND as control were scanned under isoflurane anesthesia (1.5%–4.0%) with a high‐resolution μCT system (vivaCT40; Scanco Medical), according to the current guidelines (Bouxsein et al., 2010). Images with an isotropic voxel size of 10.5 μm were acquired from 1000 projections done with an X‐ray tube voltage of 70 kVp, a current of 114 μA, and an integration time of 300 ms. Proximal tibiae were analyzed using a 700‐μm‐high volume of interest (VOI) including trabecular bone beginning immediately below the most distal part of the growth plate and a 500 μm‐high VOI for evaluation of the cortical parameters starting 1150 μm under the growth plate.

#### 4.6.2 BMAT evaluation

For assessment of the BMAT in the tibiae, we used contrast‐enhanced μCT (CE‐CT). For this, tibiae were fixed in formalin for 24 h, were transferred to PBS, and were thereafter transferred to a polyoxometalate (POM)‐based contrast solution (35 mg of Hafnium‐Wells Dawson POM per 1 ml PBS). Prior to the POM‐staining, the distal ends of the bones were removed to allow better diffusion of the contrast solution into the bone marrow compartment. Samples were incubated in the contrast solution, while shaking gently, for 48 h prior to CE‐CT imaging. We used a Phoenix NanoTom M (GE Measurement and Control solutions) at a voltage of 60 kV and a current of 140 μA, and a 0.2 mm aluminum filter was used. The exposure time was 500 ms, and 1200 images were acquired over 360° (frame averaging = 3; image skip = 1). We scanned at a 2 μm isotropic voxel size. The BMAT volume was assessed in the proximal metaphysis starting directly underneath growth plate and covering a height of 1.2 mm distal to the growth plate using CTAn (Bruker MicroCT), as previously described (Kerckhofs et al., 2018).

### 4.7 Biochemical markers of bone turnover assays

Mouse CTX‐1 EIA for the quantitative determination of the C‐telopeptide of type I collagen for bone resorption and Mouse P1NP EIA for the determination of the N‐terminal propeptide of type I procollagen (P1NP) for bone formation (Immunodiagnostic Systems Nordic) were measured in serum samples from fasted animals at the end of the experiment. Two types of data were reported, bone turnover markers solely and a ratio: bone turnover markers over Bone Surface (mm^2^) as determined by μCT to normalize according to the drastic bone loss happening in some of the experimental groups.

### 4.8 RNA extraction and real‐time qPCR

RNA was extracted from cultured cells, flushed bone marrow, or homogenized bone using Trizol combined with the Qiagen Rneasy Mini Kit (Qiagen) and reverse‐transcribed using a Revert Aid H Minus First Strand cDNA Synthesis Kit (Thermo Scientific). Quantitative real‐time PCR was performed with an Applied Biosystems 7500 Real‐Time PCR System using Fast SYBR Green Master Mix (Applied Biosystems) with specific primers (Table S1). 36B4 was used as a housekeeping gene for normalization of gene expression.

### 4.9 In vitro culture of mouse bone marrow skeletal stem cells (BMSCs)

Cells were isolated as previously reported (Tencerova et al., 2018). Briefly, front and hind limbs were collected after 12 weeks of diet intervention, crushed, and digested with 5% collagenase (StemCell). After filtration and washing steps with PBS/2%FBS, BMSCs were obtained as negative fraction from triple staining with antibodies against CD45, CD31, and Ter119 (Miltenyi Biotec) and passing through the magnetic columns. BMSCs were cultivated and expanded using standard tissue culture conditions and alpha‐MEM supplemented with 20% FBS, 1% Penicilin‐Strepotomycin 1% Glutamax, non‐essential amino acids and pyruvate sodium (Gibco). At passage 3, cells were used for the subsequent molecular and cellular analyses.

### 4.10 Western blot analysis

BMSCs cells were seeded and at 80%–90% confluence and were starved in serum reduced medium (alpha‐MEM with 0.5% BSA) for 4 h prior to treatment with 100 nM insulin for 15 min. Protein lysates were prepared using protein lysis buffer including protease inhibitors (Sigma‐Aldrich). Protein concentration was measured using BCA assay (Thermo Scientific). Protein extracts (15 μg of protein) were examined by protein immunoblot analysis probing with antibodies against total AKT (#9272, Cell Signaling), pSer473AKT (#4051, Cell Signaling), β‐Actin (A2066, Sigma‐Aldrich). Immunocomplexes were detected by enhanced chemiluminescence and analyzed by Image Lab software (BioRad).

### 4.11 Analyses of global gene expression by RNA‐seq

RNA‐seq was performed according to manufacturer's instructions (TruSeq 2, Illumina) using 1 μg RNA for preparation of cDNA libraries. Sequencing reads were mapped to the mouse genome (mm10) using STAR (Dobin et al., 2013) and tag counts were summarized at the gene level using HOMER (Heinz et al., 2010). TiCoNE (Wiwie et al., 2019) was used to cluster differentially expressed genes as determined by DESeq2 (Love et al., 2014), using a cutoff of FDR < 0.05. Gene ontology analysis was performed using GOseq (Young et al., 2010) with all detected genes as background and pathway enrichment analysis was done using a hypergeometric test with gene sets from the SPEED pathway (Parikh et al., 2010).

### 4.12 Liquid chromatography high‐resolution mass spectrometry (LC–MS)‐based metabolomics

#### 4.12.1 Metabolite extraction

Femur were collected, cleaned of soft tissue then flash frozen in liquid nitrogen. Each femur was crumbed and grounded into powder in liquid nitrogen. Metabolites were extracted in cold (−20°) with extraction solvent (50% methanol, 30% acetonitrile, and 20% water), 10 μl per mg tissue. Samples were then homogenized by sonication in a Biorupter sonicator (10 cycles, 30 s, high power) and mixed in a thermomixer at 4°C before a 30 min incubation at −20°C. Cell debris and proteins were removed from the samples by 15 min centrifugation at 16,000 *g* at 4°C. At last, supernatant were lyophilized and resuspended in 30 μl 1% formic acid of which 8 μl were transfers to a pooled (quality control) sample.

#### 4.12.2 Liquid chromatography high‐resolution mass spectrometry

The samples were overall analyzed as in Dall et al. (2021). In brief, 5 μl was injected on a Vanquish Horizon UPLC (Thermo Fisher Scientific) where a flow of 400 μl/min was used with the following composition of eluent A (0.1% formic acid) and eluent B (0.1% formic acid, acetonitrile): 3% B from 0 to 1.5 min, 3% to 40% B from 1.5 to 3 min, 40% to 95% B from 3 to 5 min, 95% B from 5 to 7.6 min and 95% to 3% B from 7.6 to 8 min before equilibration for 3.5 min with the initial conditions. The flow from the LC coupled to a Q Exactive HF mass spectrometer (Thermo Fisher Scientific) for mass spectrometric analysis in both positive and negative ion modes.

#### 4.12.3 Data processing

The raw data were also processed as in Dall et al. (2021) using MZmine (v 2.53; Pluskal et al., 2010). Features were annotated by searching against NIST17 Tandem Mass Spectral Library, MoNA – MassBank of North America library and finally SIRUIS (v. 4.7.0 [Duhrkop et al., 2019]). Signals were finally corrected for drift in statTarget software (Luan et al., 2018).

### 4.13 Statistical analysis

Data were analyzed with graph pad Prism 8 software. Results are expressed as mean ± SEM. The differences between experimental groups (ND‐SHAM, ND‐OVX, HFD‐SHAM, and HFD‐OVX) were determined by one‐way ANOVA followed by Tukey post hoc test. *p* value <0.05 was considered statistically significant.

## AUTHOR CONTRIBUTIONS

DA and FF contributed equally to this work. DA and FF involved in design, conception, data generation, data acquisition, data interpretation, and manuscript writing. AC involved in bioinformatic data analysis and interpretation. ND involved in animal experiments. CS, GK, JH, NF involved in data acquisition. AR involved in bioinformatic data analysis and interpretation and manuscript revision. MT involved in design, conception, and manuscript revision. MK involved in conception, supervision of the work, data interpretation, manuscript writing and revision.

## CONFLICT OF INTEREST

The authors declare that there is no conflict of interest.

## Supporting information


Figure S1

Figure S2

Figure S3

Figure S4

Figure S5

Table S1

Table S2

Table S3

## Data Availability

RNA sequencing and processed data have been deposited in the Gene Expression Omnibus (GEO) database, accession code number is GSE194075. Metabolomics raw data are available on Mendeley database at doi: 10.17632/3jyb26mz62.1. Primary BMSCs (RNAseq) and femurs (Metabolomics) were obtained from female C57BL/6J mice at 5 months of age fed with normal or HFD and submitted or not to ovariectomy. All experimental procedures were approved by the Danish Animal Ethical committee (2017‐15‐0201‐01210).
