# Supplementary figures and images for: High‐fat diet‐induced obesity augments the deleterious effects of estrogen deficiency on bone: Evidence from ovariectomized mice
Source: Aging Cell. 2022 Oct 10;21(12):e13726. doi: 10.1111/acel.13726 (PMC9741509; doi:10.1111/acel.13726)

Supplementary Figure 1

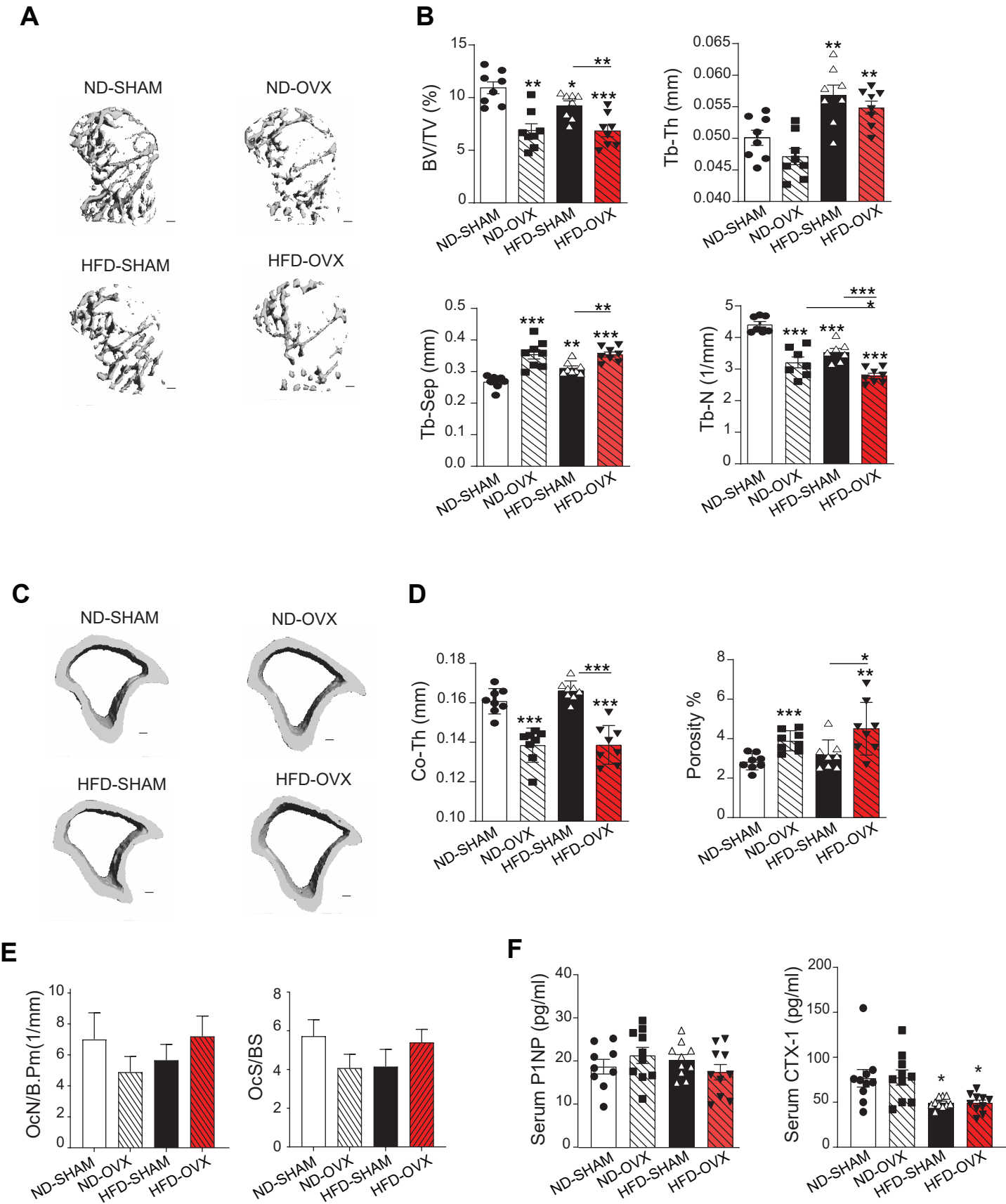

Supplement: Supplementary file 1 — Figure S1 [file ACEL-21-e13726-s004.pdf]

Supplementary Figure 2

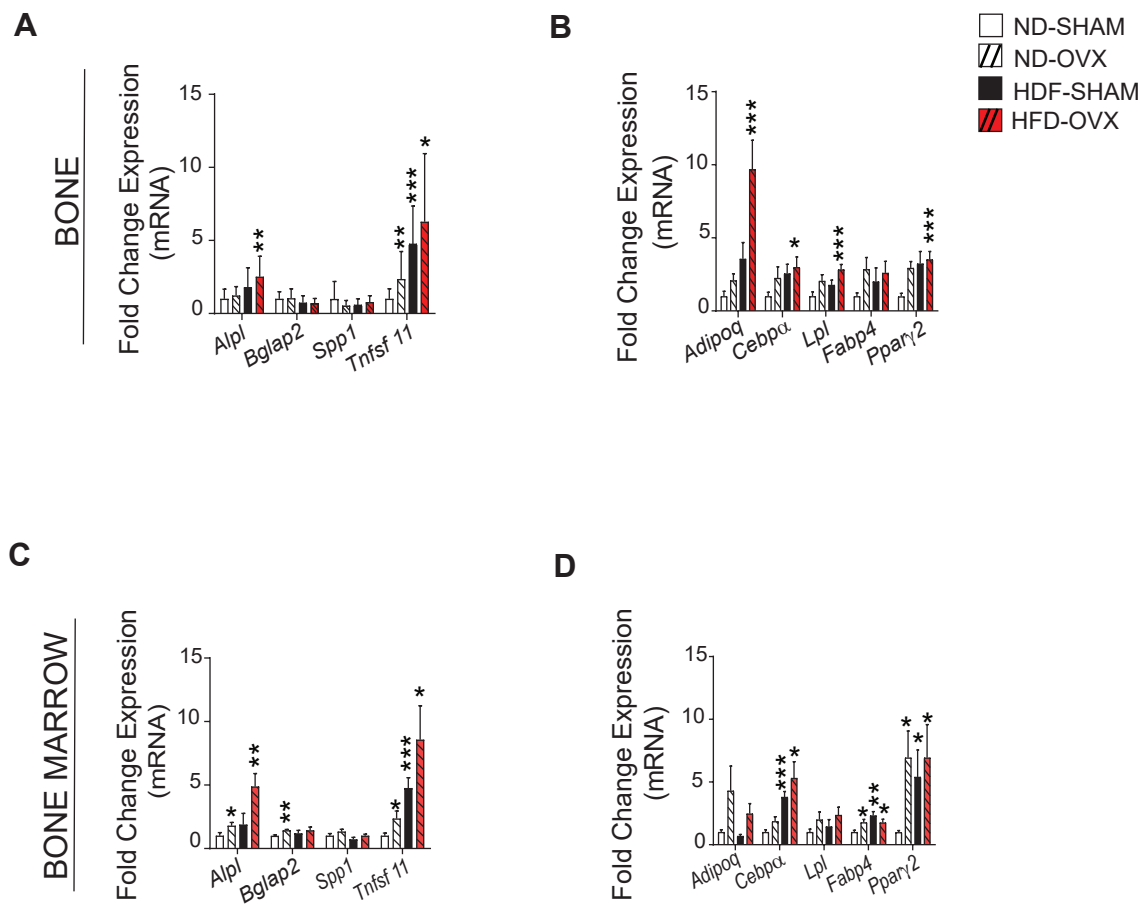

Supplement: Supplementary file 2 — Figure S2 [file ACEL-21-e13726-s008.pdf]

Supplementary Figure 3

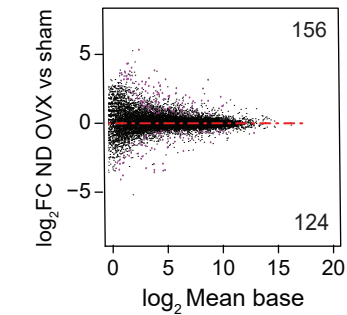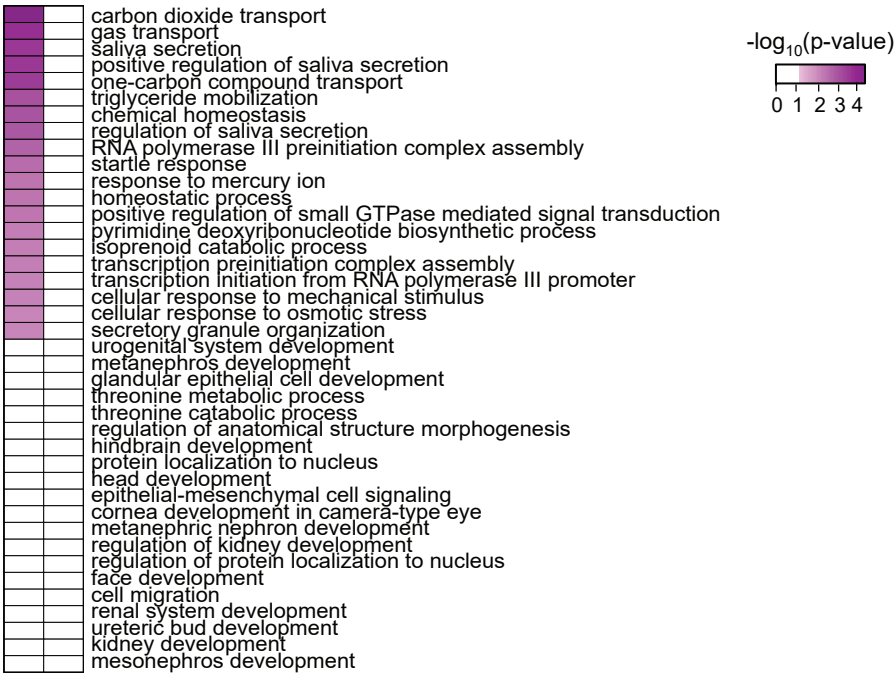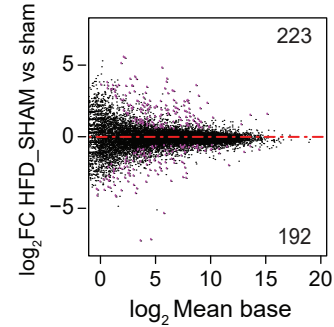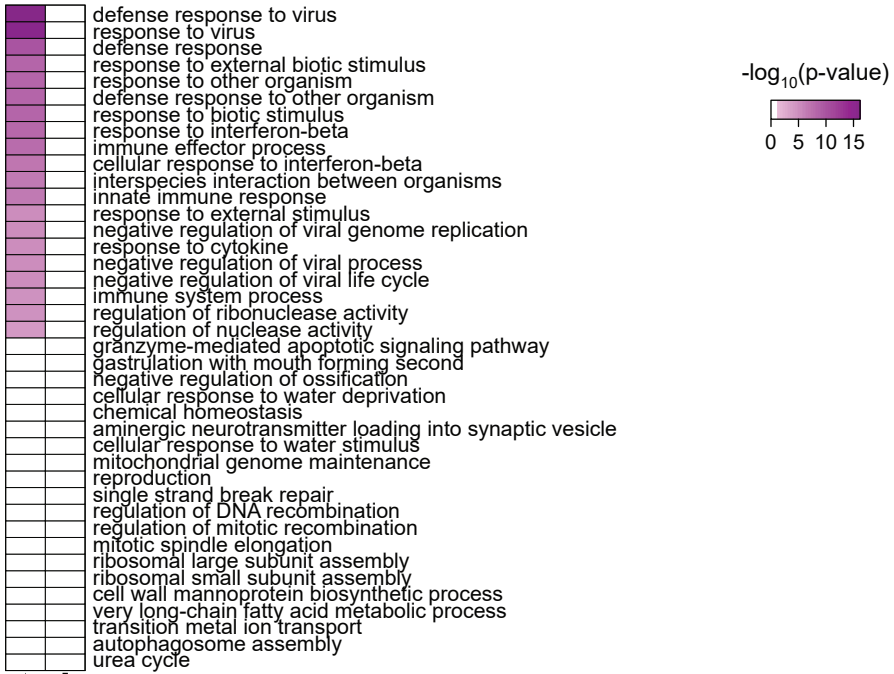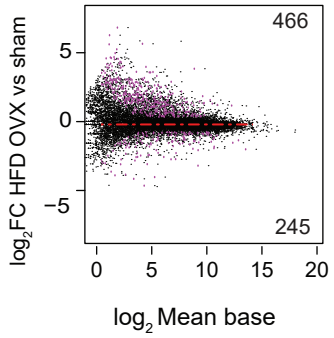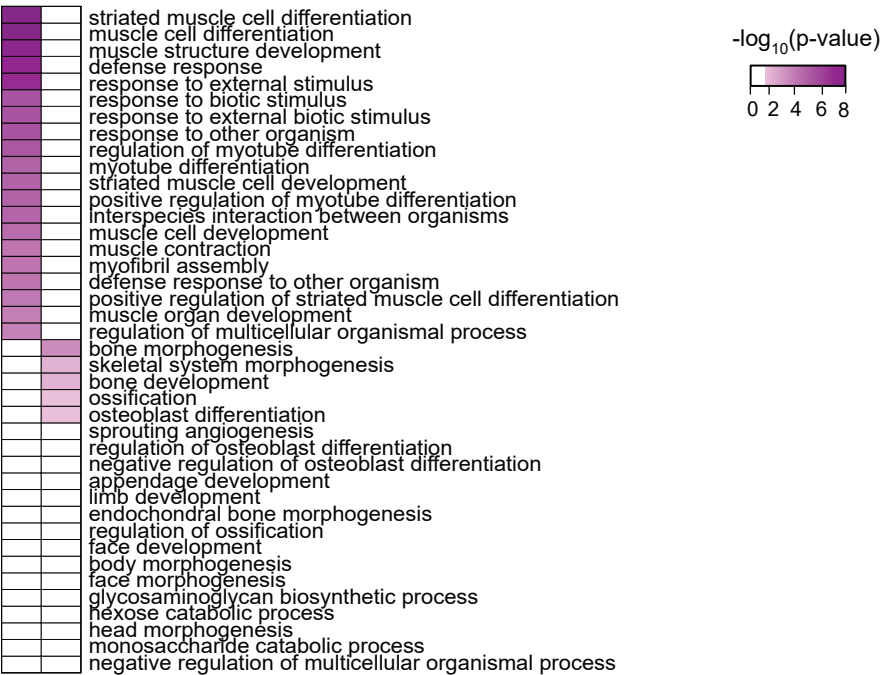

Supplement: Supplementary file 3 — Figure S3 [file ACEL-21-e13726-s005.pdf]

Supplementary Figure 4

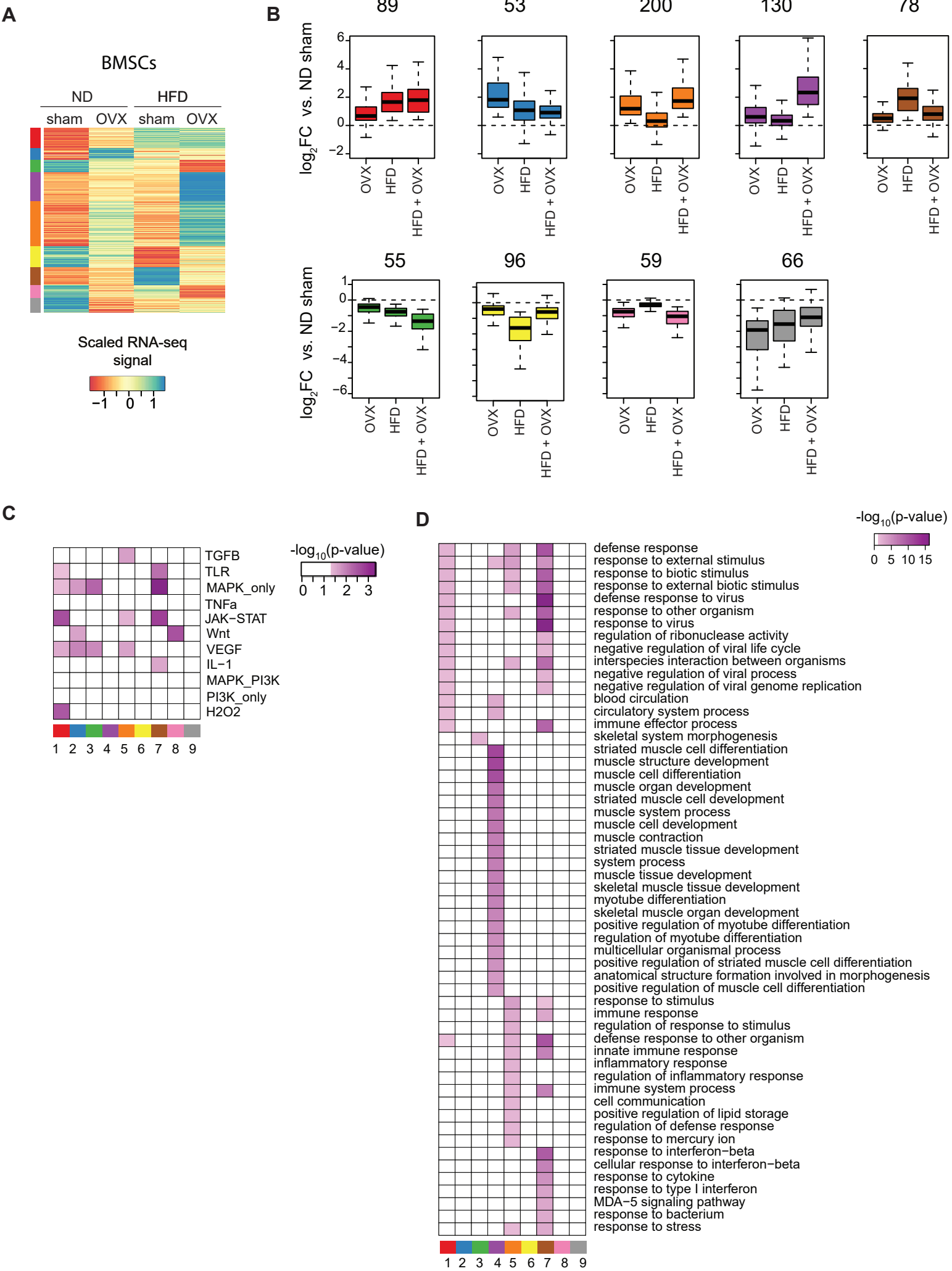

Supplement: Supplementary file 4 — Figure S4 [file ACEL-21-e13726-s003.pdf]

Supplementary Figure 5

A

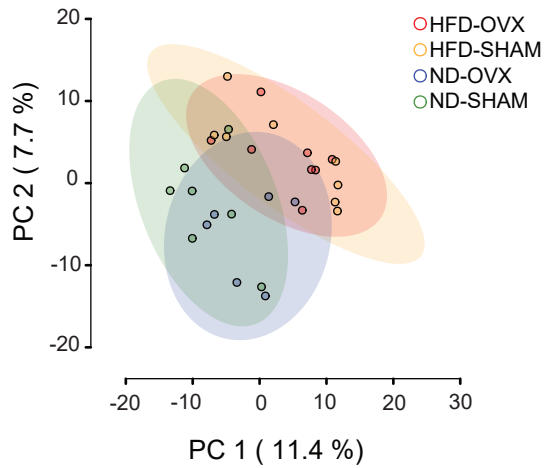

B

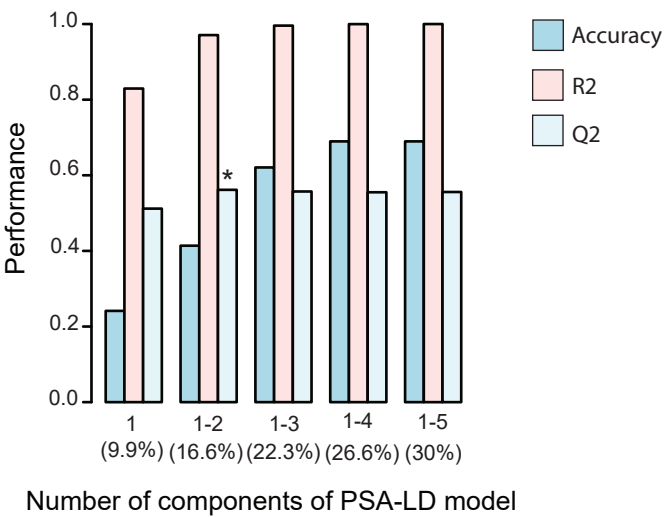

Supplement: Supplementary file 5 — Figure S5 [file ACEL-21-e13726-s006.pdf]
