# Supplementary material for: High‐fat diet‐induced obesity augments the deleterious effects of estrogen deficiency on bone: Evidence from ovariectomized mice
Source: Aging Cell. 2022 Oct 10;21(12):e13726. doi: 10.1111/acel.13726 (PMC9741509; doi:10.1111/acel.13726)
Supplement: Supplementary file 6 — Table S1 [file ACEL-21-e13726-s007.docx]

| **No** | **Name** | **Formula** | **m/z** | **Adduct** | **p.value** | **FDR** | **Pathway** |
| --- | --- | --- | --- | --- | --- | --- | --- |
| 1 | 4-Acetamidobenzoic acid | [C_9_H_9_NO_3_](https://pubchem.ncbi.nlm.nih.gov/#query=C9H9NO3) | 178.0508 | [M-H]- | 3E-06 | 0.0015 | Folate biosynthesis (linked with purine metabolism)  <https://www.genome.jp/pathway/map00790+C00568> |
| 2 | 2-(4-Acetamido-N-[2-(benzotriazol-1-yl)acetyl]anilino)-N-(2-methylbutan-2-yl)-2-(1-methylpyrrol-2-yl)acetamide | [C_28_H_33_N_7_O_3_](https://pubchem.ncbi.nlm.nih.gov/#query=C28H33N7O3) | 516.2728 | [M + H]+ | 2E-05 | 0.005 | Tryptophan metabolism  <https://www.genome.jp/pathway/map00380+C02693>  Gradual impact |
| 3 | [Benzeneacetamide, N-(2-((4-chloro-2-(2-chlorobenzoyl)phenyl)methylamino)-2-oxoethyl)-](https://www.ncbi.nlm.nih.gov/pcsubstance/?term=%22Benzeneacetamide%2C%20N-(2-((4-chloro-2-(2-chlorobenzoyl)phenyl)methylamino)-2-oxoethyl)-%22%5bCompleteSynonym%5d%20AND%203050100%5bStandardizedCID%5d) | [C_24_H_20_Cl_2_N_2_O_3_](https://pubchem.ncbi.nlm.nih.gov/#query=C24H20Cl2N2O3) | 455.0902 | [M + H]+ | 5E-05 | 0.0081 | NA |
| 4 | Uric acid | C5H4N4O3 | 335.0491 | [2M-H]- | 9E-05 | 0.0123 | Purine metabolism  <https://www.genome.jp/pathway/map00230> |
| 5 | 5-Hydroxy-3,4-dihydro-2(1H)-quinolinone | [C_9_H_9_NO_2_](https://pubchem.ncbi.nlm.nih.gov/#query=C9H9NO2) | 162.0559 | [M-H]- | 0.0001 | 0.016 | Folate biosynthesis (linked with purine metabolism)  <https://www.genome.jp/pathway/map00790+C00568>  Gradual impact |
| 6 | Guanosine 5'-monophosphate | [C_10_H_14_N_5_O_8_P](https://pubchem.ncbi.nlm.nih.gov/#query=C10H14N5O8P) | 364.0652 | [M+H]+ | 0.0003 | 0.0251 | Purine metabolism  Gradual impact |
| 7 | AZD8848 | [C_29_H_43_N_7_O_5_](https://pubchem.ncbi.nlm.nih.gov/#query=C29H43N7O5) | 570.3407 | [M + H]+ | 0.0004 | 0.0291 | Oligoribonucleotide |
| 8 | L-3-Aminoisobutyric acid | [C_4_H_9_NO_2_](https://pubchem.ncbi.nlm.nih.gov/#query=C4H9NO2) | 102.0559 | [M-H]- | 0.0004 | 0.0291 | Pyrimidine metabolism  Gradual impact |
| 9 | L-Propionylcarnitine | [C_10_H_19_NO_4_](https://pubchem.ncbi.nlm.nih.gov/#query=C10H19NO4) | 218.1387 | [M+H]+ | 0.0005 | 0.0291 | Lipid metabolism |
| 10 | Uric acid | C5H4N4O3 | 167.0209 | [M-H]- | 0.0006 | 0.0335 | Purine metabolism  <https://www.genome.jp/pathway/map00230> |
| 11 | 4-Amino-3,5-dichloro-alpha-[[[6-[2-(2-pyridinyl)ethoxy]hexyl]amino]methyl]benzenemethanol | [C_21_H_29_Cl_2_N_3_O_2_](https://pubchem.ncbi.nlm.nih.gov/#query=C21H29Cl2N3O2) | 426.1696 | [M + H]+ | 0.0009 | 0.0402 | NA |
| 12 | 1,1'-((2,7-Di-4-morpholinyl-6-phenyl-4-pteridinyl)imino)bis-2-propanol | [C_26_H_35_N_7_O_4_](https://pubchem.ncbi.nlm.nih.gov/#query=C26H35N7O4) | 510.2834 | [M + H]+ | 0.0009 | 0.0402 | Morpholines?? |

Supplementary Table 1 : differentially expressed metabolites in ND-SHAM, ND-OVX, HFD-SHAM and HFD-OVX. ANOVA.
