# Supplementary material for: High‐fat diet‐induced obesity augments the deleterious effects of estrogen deficiency on bone: Evidence from ovariectomized mice
Source: Aging Cell. 2022 Oct 10;21(12):e13726. doi: 10.1111/acel.13726 (PMC9741509; doi:10.1111/acel.13726)
Supplement: Supplementary file 7 — Table S2 [file ACEL-21-e13726-s001.pdf]

Normal Diet - R70

Ingredients:
Oatmeal, barley, wheat bran, wheat flour, beet fiber, potato protein, minerals, lysine, vitamins and trace elements.

Nutritional value %:
Crude protein 14.5
Crude fat 4.5
of which linoleic acid 1.0
Carbohydrate 60.1
Fiber 4.9
Ash 5.0
Water <11
Average available energy kJ/100g 1254
(calculated content)

Minerals % *:*
Calcium 0,7
Phosphorus 0,5
sodium chloride 0,4
Magnesium 0,25
Potassium 0,8

Trace elements mg/kg:
Cupper 12
Iron 145
Manganese 68
Zinc 80
Iodide 1,3
Cobalt 0,5
Selenium 0,1

Vitamins (added amount)

A 9 000 IU/kg
D 1 000 IU/kg
E 50 mg/kg
K3 15 mg/kg
B1 6 mg/kg
B2 5 mg/kg
B6 5 mg/kg
B12 0,07 mg/kg
Biotin 0,4 mg/kg
Folic acid 1,0 mg/kg
Calcium pantotenate 15 mg/kg
Niacin 20 mg/kg

Aminoacids% (calculated contents)
Lysine 0,8 (here of 0,3% addded)
Methionine 0,3
Cysteine 0,2
Threonine 0,5
Arginine 0,7
Histidine 0,5
Valine 0,6
Isoleucine 0,6
Leucine 0,9
Tyrosine 0,4
aspartic acid 0,9
Serine 0,7
Glutamic acid 3,1
Proline 1,1
Glycine 0,7
Alanine 0,7
