# Supplementary material for: High‐fat diet‐induced obesity augments the deleterious effects of estrogen deficiency on bone: Evidence from ovariectomized mice
Source: Aging Cell. 2022 Oct 10;21(12):e13726. doi: 10.1111/acel.13726 (PMC9741509; doi:10.1111/acel.13726)
Supplement: Supplementary file 8 — Table S3 [file ACEL-21-e13726-s002.pdf]

Supplementary Table 3

Hight Fat Diet (12492- Rodent diet with 60% fat).

Class Description	Ingredients		
Protien	Casein, Lactic, 30 Mesh	200	g
Protein	Cystine, L	3,0	g
Carbohydrates	Lodex 10	12	g
Cabohydrates	Sucrose, Fine Granulated	72,80	g
Fiber	Solka Floc, FCC200	50	g
Fat	Lard	245	g
Fat	Soybean oil, USP	25	g
**Minerals**		50	g
	Potassium Citrate Monohydrate		
	Calcium Phosphate, Dibasic		
	Calcium Carbonate light, USP		
	Sodium Chloride		
	Magnesium Sulfate, Heptahydrate		
	Magnesium Oxide, Heavy, DC, USP		
	Ferric Citrate		
	Manganese Carbonate Hydrate		
	Zinc CarbonateChromium Potassium Sulfate		
	Chromium Potassium Sulfate		
	Copper Carbonate		
	Ammonium Molybdate Tetrahydrate		
	Sodium Flouride		
	Sodium Selenite		
	Potassium Iodate		
Vitamin		1	g
	Vitamin E Acetate, 50%		
	Niacin (B3)		
	Biotin, 1%		
	Pantothenic acid, d, Calcium B5		
	D3 100,000 IU/gm		
	B12, 0.1% Mannitol		
	A Acetate, 500,000IU/gm		
	Pyridoxine Hcl, B6		
	Riboflavin, B2		
	Thiamine Hcl, B1		
	Folic acid		
	Menadione Sodium Bisulfite		
Dye	Dye, Blue FD&C #1, Alum. Lake 35-42%		

Total : 773,85 g

https://researchdiets.com/formulas/d12492
